# Use of data mining algorithms in prediction of eggshell thickness from egg quality traits of Potchefstroom Koekoek layers

**DOI:** 10.1038/s41598-025-86356-6

**Published:** 2025-01-11

**Authors:** Kagisho Madikadike Molabe, Thobela Louis Tyasi, Vusi Gordon Mbazima

**Affiliations:** 1 Department of Agricultural Economics and Animal Production, University of Limpopo, Private Bag X1106, Sovenga, 0727 South Africa; 2 Department of Biochemistry, Microbiology &Biotechnology, University of Limpopo, Private BagX1106, Sovenga, Limpopo, 0727 South Africa

**Keywords:** Eggshell thickness, CART, CHAID, MARS, Ex-CHAID, Genetics, Zoology

## Abstract

Egg quality is affected by lot of factors. Study was conducted to compare performance of data mining algorithms; Classification and regression tree (CART), Chi-square automatic interaction detection (CHAID), Exhaustive chi-square automatic interaction detection (Ex-CHAID) and Multivariate adaptive regression spline (MARS) in prediction of Potchefstroom Koekoek’s eggshell thickness from egg quality traits. 350 eggs were collected at 31st to 39th week to examine the egg quality traits. MARS with R^2^(0.86) revealed yolk ratio, shell weight, egg shape index, yolk ratio, shell ratio, albumen weight and albumen ratio as explanatory variables predicting eggshell thickness. CART with R^2^ (0.37), yolk/albumen ratio was noted to be influential predictor of eggshell thickness. CHAID and Ex-CHAID (R^2^= 0.35) discovered egg weight as the best predictor of eggshell thickness. MARS with R^2^(0.86) revealed yolk ratio, shell weight, egg shape index, yolk ratio, shell ratio, albumen weight and albumen ratio as explanatory variables predicting eggshell thickness. MARS had high r (0.925), R^2^ (0.856) and lower RMSE (0.129) and AIC (-975.331) compared to CHAID, Ex-CHAID and CART leading MARS to be the best data mining algorithm when predicting the eggshell thickness using egg quality traits.

## Introduction

Poultry industry as one of agricultural sector contributes to the economy through provision of food such as meat and eggs contributing 60% of protein to human’s diet^1,2^. Indigenous chickens due to their low maintenance, disease resistance and adaptability to harsh climatic conditions, their production increase daily^3^. As explained by Tyasi et al.^4^, Potchefstroom Koekoek chicken breed is an indigenous chicken derived from crossing White Leghorn, the Black Australorp, and the Barred Plymouth Rock and it’s characterised by black and white colour patterns. Potchefstroom Koekoek chicken breed is a prolific brown shelled egg layer with average weight of 55.7 g, brown egg shell results from syntheses of protoporphyrin IX pigment in the shell gland which is deposited onto all shell layers including the shell membranes, but most the calcareous shell and in the cuticle^5^. Egg quality is affected by lot of factors including genes and have impact on the acceptability by consumers and recently, the genes that are involved in pigment synthesis have been identified, but the genetic control of synthesis and deposition of brown pigment is not fully understood^6^. Literature on metabolic pathway of pigments in the shell gland and the genes which overexpression can significantly increase the intensity shell pigment and improve shell quality. Egg quality traits are constituents of the egg and are divided into internal, focusing on the content of the egg and external, focusing on the eggshell egg quality traits^4^. Eggshell as the closure and the protector of the internal content of the egg plays a major role in protecting the embryo from external forces and enabling development^7^. However, Potchefstroom Koekoek layers experience lot of damage and loss resulting from cracked eggshell due to diseases, infections, inadequate nutrition, expression of genes and environmental factors^8^. Egg quality has long been subjected to genetic selection, and considerable improvement of eggshell quality has been reported using linear and multiple regression^9^. Recently the approach of data mining algorithm gained exposure in the poultry industry where they are utilised to predict traits of economic importance such as egg weight^10^. In making decisions for selection to improve desired traits, breeders deal with a lot of data consisting of a lot of factors which in most cases renders the data multidimensional.

Data mining algorithms are more considered as they possess in multi-collinearity^11^. Additionally, as documented by Kantardzic^12^, these algorithms are computer-based method to discover information from data. To the best of authors’ knowledge, importance of the data mining algorithms in poultry industry had been poorly utilised hence the aim of the current study was to compare performance of data mining algorithms; Classification and regression tree (CART), Chi-square automatic interaction detection (CHAID), Exhaustive chi-square automatic interaction detection (Ex-CHAID) and Multivariate adaptive regression spline (MARS) in prediction of Potchefstroom Koekoek eggshell thickness from egg quality traits; Albumen weight (AW), Albumen ratio (AR), Yolk weight(YW), Yolk ratio (YR), Yolk/albumen ratio (YAR), egg weight (EW), egg length (EL), egg width (EWDT), egg volume (EV) and egg shape index (ESI), shell weight (SW), shell ratio (SR), shell strength (SS), shell surface area (SSA), unit surface shell weight (USSW) and eggshell thickness (STA).

Reports from the study might help Potchefstroom Koekoek layers in improving the eggshell through selection of accurate data mining algorithm and selection of best traits in predicting eggshell thickness for improvement during breeding. This study will also increase the employability of data mining algorithms in the poultry industry.

## Materials and methods

### Study area

The research was conducted at the University of Limpopo experimental farm, located in Mankweng village within Capricorn District of Limpopo Province, South Africa. The farm lies at latitude of 27.55 ºSouth and longitude 24.77 ºEast and experiences semi-arid climatic conditions^13^.

### Experimental animal, management, design

A total of one hundred (*n* = 100) point of lay Potchefstroom Koekoek chicken layers were bought from local market were used as experimental animals. Animals were raised under intensive management system, they were housed in cages (medium-tier cage system with 3 hens in each cage of 30 × 30 × 25.5 cm) in a house that was cleaned with water, disinfectant and left for 14 days to destroy any remaining organisms. Water and feeds (layers’ mash containing 16% crude protein and 11.97 MJkg/DM) and water were always provided. Standard management protocol was followed as suggested by Manyelo et al.^13^. Complete randomise design was applied as the research design.

### Egg collection

A total of three hundred and fifth (*n* = 350) eggs were randomly collected from week 31st to 39th, numbered correctly and taken to laboratory for measurements of egg characteristics.

### Collection of internal egg quality traits

Albumen weight (g), Albumen ratio (%), Yolk weight(g), Yolk ratio (%), Yolk/albumen ratio (%) were measured as internal egg quality trait. The yolk and albumen were allowed to pass through a whole created on the eggs and they were separated using egg yolk separator. The albumen weight was determined by subtracting yolk weight (measured using electronic scale of crate scale 300 kg x 01 kg of Tronic services, South Africa) from shell weight. Other internal egg quality traits were determined following Eqs. (1, 2 and 3) by Ashraf et al.^14^.

1 $$\:\text{A}\text{l}\text{b}\text{u}\text{m}\text{e}\text{n}\:\text{r}\text{a}\text{t}\text{i}\text{o}\:\left(\text{\%}\right)=\frac{\text{a}\text{l}\text{b}\text{u}\text{m}\text{e}\text{n}\:\text{w}\text{e}\text{i}\text{g}\text{h}\text{t}}{\text{e}\text{g}\text{g}\:\text{w}\text{e}\text{i}\text{g}\text{h}\text{t}}\times\:100$$

2 $$\:\:Yolk\:ratio\:\left(\%\right)=\frac{\text{y}\text{o}\text{l}\text{k}\:\text{w}\text{e}\text{i}\text{g}\text{h}\text{t}}{\text{e}\text{g}\text{g}\:\text{w}\text{e}\text{i}\text{g}\text{h}\text{t}}\times\:100$$

3 $$\:\:\:Yolk/Albumen\:\left(\%\right)\:=\frac{\text{y}\text{o}\text{l}\text{k}\:\text{w}\text{e}\text{i}\text{g}\text{h}\text{t}}{\text{a}\text{l}\text{b}\text{u}\text{m}\text{e}\text{n}\:\text{w}\text{e}\text{i}\text{g}\text{h}\text{t}}\times\:100$$

### Collection of external egg quality traits

The external egg quality traits collected were egg weight (g), egg length (mm), egg width (mm), egg volume (cm^3^) and egg shape index (%). Egg weight was weighed using balanced weighing scale (crate scale 300kgx01kg) of Tronic services, South Africa. Egg length (mm) and egg width (mm) were measured using vernier caliper. The shape index was determined using Eq. (4) as described by Ashraf et al.^14^whereases egg volume (cm^3^) was determined following Eq. (5) by Kgwatalala et al.^15^.

4 $$\:Shape\:index\:\left(\%\right)=\frac{egg\:width}{egg\:length}\times\:100$$

5 $${\text{Egg volume}} = [0.6057- (0.018 - {\text{Egg width}} * {\text{Egg length}} * {\text{egg width}}^2)].$$

### Collection of eggshell quality traits

The shell weight (g/cm2), shell ratio (%), shell strength (N), shell surface area (cm^3^), unit surface shell weight (g/cm^2^) and eggshell thickness (mm) were measures. Shell weight was measured using electronic scale of scale 300 kg x 01 kg from Tronic services, South Africa. Three regions (sharp, equatorial, and blunts) of the eggshell were measured using micrometer gauge (QCT shell thickness micrometer, TSS, England) after exposure to air for 72 h to get eggshell thickness, the average was considered. Eggshell strength (N) was measured using QCT shell strength tester. Other shell quality trait was measured following Eq. (6) by Ashraf et al.^14^.

6 $$\:\text{S}\text{h}\text{e}\text{l}\text{l}\:\text{r}\text{a}\text{t}\text{i}\text{o}\:\left(\text{\%}\right)=\frac{\text{s}\text{h}\text{e}\text{l}\text{l}\:\text{w}\text{e}\text{i}\text{g}\text{h}\text{t}}{\text{e}\text{g}\text{g}\:\text{w}\text{e}\text{i}\text{g}\text{h}\text{t}}\times\:100$$

### Classification and regression tree (CART) data mining algorithm

The CART is a decision tree which split data into uniform subgroups with regards to the reliant variable^16^. Decision tree is constructed partitioning a node 0 (parent note) which contains all data set into chid notes ensuring minimum error variance using cross-validation training and test sets^17^. According to^18^documentation, CART algorithm, prune to avoid unnecessary nodes in the decision tree diagram and the technique aims to have a terminal node to increase proportion of differences between nodes. CART as an understandable model to predict an event on both ordinal and nominal scale^16^. The goal of this technique is to have terminal nodes to increase the proportion of differences between nodes^18^.

### Chi-squared automatic interaction detection (CHAID) data mining algorithm

The CHAID divide population into categories such that the variation in a dependent variable within groups is minimized and among groups is maximized^10^. It’s a prediction model that defines the outcome in each dependent variable, moreover ordinal, continuous and nominal data can be used^19^.

### Exhaustive CHAID (Ex-CHAID) data mining algorithm

The Ex-CHAID) is the extension of CHAID algorithm and aim is to maximize the variance between nodes, depending upon their tree depth^20^. It expresses the same splitting and stopping steps as CHAID however it has got the merging step more exhaustive than CHAID^21^.

### Multivariate Adaptive Regression Splines (MARS) data mining algorithm

The MARS data mining algorithm as a form of regression analysis developed in 1991 by Friedman^22^. MARS is a non-parametric regression procedure which can produce linear models that are nonlinearities and interactions between variables^11,23^. Its advantage is that it can overcomes the multicollinearity problems^17^. The MARS algorithm was conducted as explained by^24^, and its prediction equation can be written as follows:

7 $$\:f\left(x\right)={\beta\:}_{0}+\sum\:_{m=1}^{m}{\beta\:}_{m}{\lambda\:}_{m}\left(x\right)$$

where f(x) is the expected response, β_0_ and β_m_ are parameters that are calculated to give the best data fit, and m is the number of BFs in the model. In the MARS model, the basis function composed of be a single univariable spline function or a combination of more than one spline function for diverse predictor inputs. The spline BF, λ_m_(x), is defined as:

8 $$\:{\lambda\:}_{m}\left(x\right)=\:\prod\:_{k=1}^{{k}_{m}}\left[{s}_{km}\left({X}_{v\left(k,m\right)}-{t}_{k,m}\right)\right]$$

where t_k, m_ denotes the knot location; s_km_denotes the right/left regions of the corresponding step function, taking either 1 or − 1; v(k, m) denotes the predictor variable’s label; and km is the number of knots. The pruning process was used to remove the basic functions that had a low contribution to the model fitting performance following the generalised cross-validation error (GCV)^24^:

9 $$\:GCV\left(\lambda\:\right)=\:\frac{{\sum\:}_{i=1}^{n}{\left({y}_{i}-\:{y}_{ip}\right)}^{2}}{{\left(1-\:\frac{M\left(\lambda\:\right)}{n}\right)}^{2}}$$

where n represents the number of training cases, y_i_ shows the observed value of the responsible variable, y_ip_ as the estimated value of the response variable and M(λ) represents the penalty function for the complex of the model with λ terms.

### Performance assessment of used data mining algorithms

The goodness of fit test was used to compare the prediction performance of CART, CHAID, Ex-CHAID and MARS as suggested by^11^.

Pearson’s correlation coefficient (r):

10 $$\:\text{r}=\frac{cov\left({y}_{i}{y}_{ip}\right)}{{S}_{yi}{S}_{Yip}}$$

Coefficient of Determination (R^2^):

11 $$\:\text{R}\text{s}\text{q}=1-\frac{\sum\:_{i=1}^{n}{\left({y}_{i}-{\widehat{y}}_{i}\right)}^{2}}{\sum\:_{i=1}^{n}{\left({y}_{i}-\stackrel{-}{y}\right)}^{2}}$$

Adjusted Coefficient of Determination (Adj.R^2^):

12 $$\:{\text{A}\text{d}\text{j}.\text{R}}^{2}=1-\frac{\frac{1}{n-k-1}\sum\:_{i=1}^{n}{\left({y}_{i}-{\widehat{y}}_{i}\right)}^{2}}{\frac{1}{n-1}\sum\:_{i=1}^{n}{\left({y}_{i}-\stackrel{-}{y}\right)}^{2}}$$

Akaike Information Criteria (AIC):

13 $$\:\text{A}\text{I}\text{C}=nln\left[\sum\:_{i=1}^{n}\frac{{\left({y}_{i}-{\widehat{y}}_{i}\right)}^{2}}{n}\right]+2k$$

Root-mean-square error (RMSE):

14 $$\:\text{R}\text{M}\text{S}\text{E}=\sqrt{\frac{1}{n}\sum\:_{i=1}^{n}{({y}_{i}-{\widehat{y}}_{i})}^{2}}$$

### Statistical analysis

The Statistical Package for Social Sciences (IBM SPSS, 2022) version 29.0 software was used for data analysis at significance level of 5%^25^. The data mining algorithms such as CART, CHAID, Ex-CHAID and MARS were used to achieve the objective of this study.

## Results

### CART data mining algorithm

**Fig. 1:**
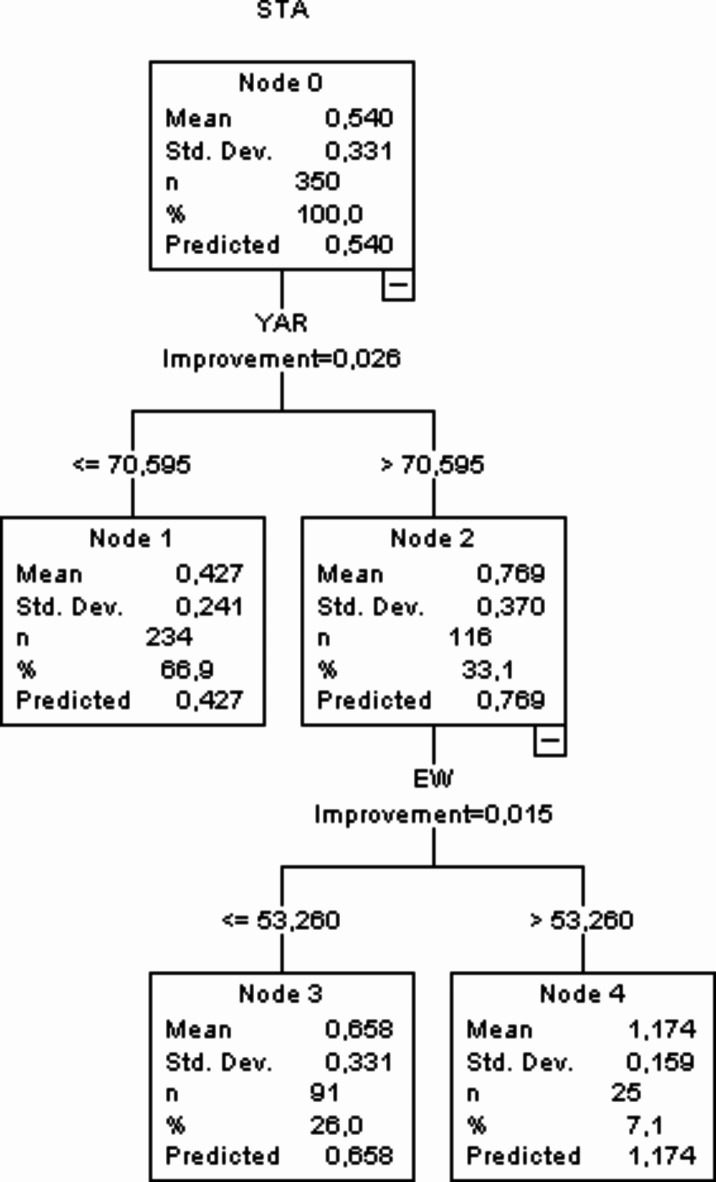
CART data mining algorithm.

Decision tree (CART) containing detailed information on the independent traits significantly affecting eggshell thickness is displayed in Fig. 1. Parent (root) node showed an average mean of shell thickness as 0.540 with standard deviation of 0.331 with total number of 350 eggs included. The root note was split into subgroups, node 1 with average mean of 0.427 and standard deviation of 0.241, node 2 with average and standard deviation of 0.769 and 0.370 respectively according to the eggshell thickness with YAR and EW as the independent traits. Node 2 was further divided to node 3 (mean of 0.658 and standard deviation of 0.331) and node 4 having a mean of 1.174 and standard deviation of 0.159 with egg weight as the independent trait. The thickest eggshell thickness mean was noted on node 4 (1.174) with YAR > 70.595 and EW > 53.260. Lowest eggshell mean was observed on node 1 (0.427) with YAR ≤ 70.595.

### CHAID data mining algorithm

**Fig. 2:**
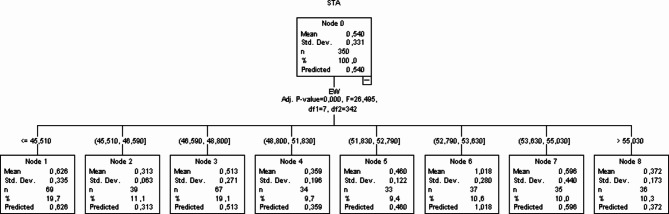
CHAID data mining algorithm.

Regression tress constructed by CHAID decision tree is displayed in Fig. 2. The overall average in the parent node with eggshell thickness as the dependent traits shown to be 0.540 with standard deviation of 0.331. Total of 350 eggs included in the study were group according to eggshell thickness and divided into subgroups called nodes. The parent node (node 0) was split to 8 nodes with EW as the independent trait.

The highest average on eggshell thickness was obtained on note 6 (1.018) with EW between 52.790 and 53.630 and the lowest average on eggshell thinness was noted on node 2 (0.313) with EW between 45.510 and 46.590.

### Ex-CHAID data mining algorithm

**Fig. 3:**
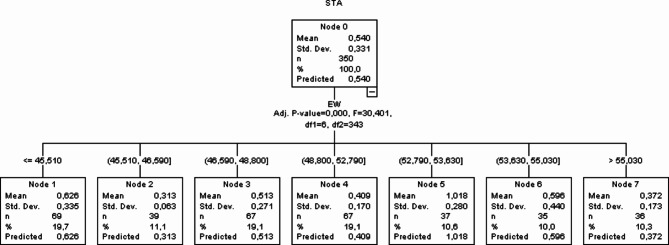
Ex-CHAID data mining algorithm.

In the Exhaustive CHAID (Ex-CHAID) in Fig. 3 noted that, an average mean (0.54) and standard deviation (0.33) were observed on node 0 with STA as the dependent trait and 350 eggs were present in the top selection generated. The node 0 was subdivide into 7 nodes based on eggshell thickness with EW as the independent trait. The lowest average on eggshell thickness was observed on note 2 (0.313) with EW between 45.510 and 46.590 and the highest average on eggshell thickness was noted on node 5 (1.018) with EW between 52.790 and 53.630.

### MARS data mining algorithm

Multivariate adaptive regression splines (MARS) algorithm reports showed that YR, SW, ESI, YR, SR, AW and AR were involved in the model (Table 1). Results showed eighteen basic functions from MARS model, three as single order term variables and fifteen as orders of interactions with an intercept of 0.307. MARS displayed positive and negative coefficient showing impact of egg quality traits on eggshell thickness. Positive influence on eggshell thickness was noted when YW < 15.53 with model coefficient of 0.372. Moreover, the model showed the effect of egg quality traits interactions on STA. Briefly, the influence on STA was 0.020 when YW > 15.53 and YR > 37.14, 0.033 when YW > 15.33 and SR > 36.3092, 0.017 when ESI < 71.8799, YW > 15.53 and YR < 37.14, 0.021 when ESI > 71.48, YW > 15.53, AR > 53.58, 0.119 when YW > 15.53, YR > 37.14, SW < 5.47 and lastly 0.016 when YW > 15.53, YR < 37.14, SR < 35.48.

**Table 1:** MARS data mining algorithm.

BF	Equation	Coefficient
Intercept		0.307
BF1	h(YW-15.53)	0.372
BF2	h(6.55-SW)	0.147
BF3	h(SW-6.55)	0.137
BF4	h(71.48-ESI) * h(YW-15.53)	−0.021
BF5	h(YW-15.53) * h(YR-37.14)	0.020
BF6	h(YW-15.53) * h(37.14-YR)	−0.059
BF7	h(YW-15.53) * h(SR-36.3092)	0.033
BF8	h(YW-15.53) * h(36.3092-SR)	−0.050
BF9	h(71.8799-ESI) * h(YW-15.53) * h(37.14-YR)	0.017
BF10	h(ESI-71.8799) * h(YW-15.53) * h(37.14-YR)	−0.011
BF11	h(ESI-71.48) * h(YW-15.53) * h(AR-53.58)	0.021
BF12	h(ESI-71.48) * h(YW-15.53) * h(53.58-AR)	−0.004
BF13	h(YW-15.53) * h(AW-27.78) * h(37.14-YR)	−0.007
BF14	h(YW-15.53) * h(27.78-AW) * h(37.14-YR)	−0.072
BF15	h(YW-15.53) * h(YR-37.14) * h(SW-5.47)	−0.044
BF15	h(YW-15.53) * h(YR-37.14) * h(5.47-SW)	0.119
BF17	h(YW-15.53) * h(37.14-YR) * h(35.48-SR)	0.016

### Data mining algorithms performance

The predictive and statistical performance using several goodness of fit criteria are presented in Table 2. MARS was estimated to have high r, R^2^, Adjusted R^2^ and lower RMSE and AIC as compared to CHAID, Ex-CHAID and CART, as such lead MARS to be the best accurate data mining algorithm when predicting the eggshell thickness using egg quality traits.

**Table 2:** Predictive performance of data mining algorithms.

Goodness of fit	CHAID	Ex-CHAID	CART	MARS	Decision
r	0.593	0.589	0.611	0.925	Greater is better
Adjusted R^2^	0.350	0.345	0.372	0.845	Greater is better
R^2^	0.352	0.347	0.374	0,856	Greater is better
RMSE	0.267	0.268	0.2625	0.129	Smaller is better
AIC	−923.889	−921.488	−936.059	−975.331	Smaller is better

## Discussion

Data mining algorithms provide reliable information on relation between traits as they cater for multi-collinearity problem among traits than regression techniques^26^. Hence the current study compares performance of data mining algorithms including CARD, CHAID, Ex-CHAID and MARS on prediction of Potchefstroom Koekoek’s eggshell thickness from egg quality traits. The output on CART data mining algorithm with R^2^ of 0.37, yolk/albumen ratio was noted to be influential predictor of eggshell thickness. Finding further showed that both CHAID and Ex-CHAID data mining algorithms (R^2^= 0.35) discovered egg weight as the best predictor of eggshell thickness. While MARS with R^2^of 0.86 revealed yolk ratio, shell weight, egg shape index, yolk ratio, shell ratio, albumen weight and albumen ratio as explanatory variables predicting eggshell thickness. There is no literature on use of data mining algorithm in prediction of eggshell from egg quality traits, however data mining algorithm had been applied and explored in the poultry industry in prediction of egg weight. Due to lack of literature on the prediction of eggshell thickness using data mining algorithms, current findings were discussed based on comparing current study results using studies conducted on predicting egg weight from egg quality traits using data mining algorithm. Study by^19^compared CHAID and CART in predicting egg weight from egg quality traits on indigenous free-range chickens and found that CHAID algorithm had the highest R^2^(0.82) and it was recommended for prediction of egg weight. Study conducted by^10^on predictive performance of some data mining algorithms (CHAID, exhaustive CHAID and CART) implemented in the estimation of egg weight in quail, exhaustive CHAID model was best in predicting egg weight with R^2^of 85.86. On the study that was conducted on relationship among egg quality traits in Japanese quails and prediction of egg weight and color using data mining algorithms, MARS performed better than CART in estimating egg weight in quails^27^. Regression techniques has been used in predicting eggshell thickness from egg quality traits. Study conducted by^28^ on Japanese Quails reported egg length as a desirable trait for accurate prediction of eggshell thickness using linear regression model. Nordstrom et al.^29^highlighted on study conducted on Single Comb White laying hens that shell thickness, is more precisely estimated from egg specific gravity when egg weight is also included as the second independent variable on multiple linear regression model. Study on Japanese Quails and documented that eggshell thickness may be predicted using egg width^30^. Current study outcomes suggests that eggshell thickness of Potchefstroom Koekoek may be predicted from egg quality traits using data mining algorithms. The study showed that MARS (R^2^ = 0.86) was satisfactory in predicting eggshell thickness of Potchefstroom Koekoek using egg quality traits. From MARS model, yolk ratio, shell weight, egg shape index, yolk ratio, shell ratio, albumen weight and albumen ratio showed contribution to eggshell thickness. Potchefstroom Koekoek farmers might use the findings from the current study to improve eggshell thickness of their chicken by selecting traits (Yolk ratio, shell weight, egg shape index, yolk ratio, shell ratio, albumen weight and albumen) that showed influence on eggshell thickness. Several studies were conducted on prediction of egg weight using data mining algorithm while prediction on eggshell thickness is neglected, we recommend that more studies should be conducted on the prediction of eggshell thickness using data mining algorithm on different chicken breeds.

## Conclusions

In the current study, data mining algorithm may be used to predict eggshell thickness using egg quality traits. MARS was estimated to have high r (0.925), R^2^(0.856), Adjusted R^2^ (0.845) and lower RMSE (0.129) and AIC (−975.331) as compared to CHAID, Ex-CHAID and CART. The study concludes that MARS data mining algorithm was more informative in the predictive accuracy of eggshell, as such lead MARS to be the best accurate data mining algorithm when predicting the eggshell thickness using egg quality traits.

### Recommendation

Therefore, it is recommended that chicken breeders may consider applying MARS algorithm for the prediction of eggshell thickness. More studies need to be done on predicting eggshell thickness in the poultry industry.

## Data Availability

The data that support the findings of this study are available from the corresponding author upon reasonable request.
